# Immunoglobulin E-Mediated Anaphylaxis to Sesame

**DOI:** 10.1097/WOX.0b013e31817eef29

**Published:** 2008-08-15

**Authors:** Emmanuella Guenova, Sonya Genova, Bogomil Voykov, Silvia Novakova, Vanyo Mitev

**Affiliations:** 1 Department of Dermatology, Eberhard Karls University, Tuebingen, Germany; 2 Department of Chemistry and Biochemistry, Medical University Sofia, Bulgaria; 3 Department of Allergology, Medical University Sofia, Bulgaria; 4 Department of Ophthalmology, Eberhard Karls University, Tuebingen, Germany

## To the Editor

The incidence of food allergy is age dependent, particularly affecting children and young patients. When self-reported, it ranges between 3% and 35%. However, placebo-controlled food challenge is positive in just 1% to 4% of all suspected cases [1]. In elderly, anaphylactic reactions tend to be less severe, and a primary manifestation at this age is unusual.

A 70-year-old white man needed twice in the past 6 months emergency medical help because of acute systemic anaphylactic reactions, both timely associated with sesame (*Sesamum indicum*) ingestion. Fifteen minutes after consumption of sesame-poppy-raisin sweets or sesame bread, the patient developed generalized urticaria with periorbital, periocular, and laryngeal edema associated with severe hypotension and unconsciousness. In both cases, emergency treatment with intramuscular administration of epinephrine and high doses of corticosteroids and antihistamines intravenously was needed. Previous allergic reactions were denied. Occasional contact to sesame throughout the life span and noncontributory further own and familial anamnesis were stated.

Skin prick tests to common inhalant allergens, house-dust mite, preservatives, and food allergens were negative. Furthermore, prick-to-prick tests with poppy seed, raisins, and wheat flour remained negative as well.

Surprisingly, skin prick-to-prick tests, both with sesame seed and sesame oil, revealed a pronounced positive reaction compared with the standard histamine control and the control reaction of a healthy volunteer. The specific immunoglobulin E (IgE) for sesame was highly positive (6.37 kUA/L, Phadia ImmunoCAP), whereas total serum IgE (93.5 kUA/L) and basal serum tryptase level (3.1 Hg/L) remained in the normal range.

Complying with the patient's wish to avoid unnecessary diet restriction, we conducted a double-blind placebo-controlled food challenge starting with 25 mg of sesame seeds in bread porridge. Fifteen minutes after a dose of 100 mg of backed sesame seed, acute disseminated urticaria, pruritus, and periorbital and periocular edema occurred without reaction of the upper or lower airways. The IgE-mediated mast cell activation was proven by increased serum tryptase level (17.5 Hg/L) 2 hours after the food challenge-induced anaphylaxis.

The increased reporting of allergic type I hypersensitivity to sesame (*S. indicum*) with major allergens Ses i 1-7 suggests its growing importance [2-4]. Several recent reports from Pajno et al[5] and Eberlein-König et al[6]describe non-IgE-mediated sesame anaphylaxis. Our case of classic IgE-mediated anaphylaxis to sesame with primary manifestation in a 70-yearold patient represents the potential life-threatening severity of food anaphylaxis, especially to foods with pronounced allergenic properties such as sesame, irrespective of the patient's age.

Emmanuella Guenova* **

**Sonya Genova**‡

**Bogomil Voykov**§

**Silvia Novakova**‡

Vanyo Mitev**

*Department of Dermatology

Eberhard Karls University

Tuebingen, Germany

Departments of **Chemistry and Biochemistry

and ‡Allergology

Medical University

Sofia, Bulgaria

and §Department of Ophthalmology

Eberhard Karls University

Tuebingen, Germany

emmanuella.guenova@med.uni-tuebingen.de
